# Correction: Land reclamation pattern and environmental regulation guidelines for port clusters in the Bohai Sea, China

**DOI:** 10.1371/journal.pone.0302709

**Published:** 2024-04-18

**Authors:** Gaoru Zhu, Zhenglei Xie, Honglei Xu, Minxuan Liang, Jinxiang Cheng, Yujian Gao, Liguo Zhang

In the Abstract section, there is an error in the fifth sentence. The correct sentence is: The reclamation area of the 13 cities in the Bohai Sea in 2002–2018 was 2,300 km2, which decreased the area of the sea by 3%.

In the Conclusion section, there is an error in the seventh sentence of the first paragraph. The correct sentence is: Findings showed that the area of reclamation of the 13 cities during 2002–2018 was 2,300 km2.
