# Supplementary material for: Land reclamation pattern and environmental regulation guidelines for port clusters in the Bohai Sea, China
Source: PLoS One. 2021 Nov 3;16(11):e0259516. doi: 10.1371/journal.pone.0259516 (PMC8565771; doi:10.1371/journal.pone.0259516)
Supplement: Attachment — Submitted filename: Response to Reviewers20210830.docx [file pone.0259516.s001.docx]

## Responses to Editor-in Chief’s recommendation

(1) Question: Thank you for submitting your manuscript to PLoS ONE. After careful consideration, we feel that it has merit but does not fully meet PLOS ONE’s publication criteria as it currently stands. Therefore, we invite you to submit a revised version of the manuscript that addresses the points raised during the review process.

Response: We are thankful to the editors and reviewers for giving constructive comments on the manuscript and **providing a merit evaluation and a revision opportunity**. The editors believe that the current version does not fully meet PLoS ONE's publication criteria. These constructive comments proposed by the reviewers are helpful to the improvement of the previous version. We have identified the reviewers' points one-by-one raised during the review process carefully according to the reviewer’s comments and we have submitted a revised manuscript. We hope that the revised manuscript would be accepted very soon.

(2) Question: Please submit your revised manuscript by Sep 23 2021 11:59PM. If you will need more time than this to complete your revisions, please reply to this message or contact the journal office at [plosone@plos.org](mailto:plosone@plos.org). When you're ready to submit your revision, log on to https://www.editorialmanager.com/pone/ and select the 'Submissions Needing Revision' folder to locate your manuscript file.

Please include the following items when submitting your revised manuscript:

- A rebuttal letter that responds to each point raised by the academic editor and reviewer(s). You should upload this letter as a separate file labeled 'Response to Reviewers'.
- A marked-up copy of your manuscript that highlights changes made to the original version. You should upload this as a separate file labeled 'Revised Manuscript with Track Changes'.
- An unmarked version of your revised paper without tracked changes. You should upload this as a separate file labeled 'Manuscript'.

Response: We are thankful for you providing a revision opportunity to our manuscripts and we will resubmit the revised manuscript before the due time based on the above website. The file labeled 'Response to Reviewers' has been uploaded as separate file and has been responded to each point raised by the academic editor and reviewers. Moreover, we have also submitted a separate file labeled "Revised Manuscript with Track Changes", which has highlighted changes made to the original version. The unmarked version of revised paper without tracked changes has been also uploaded as separated file. Please refer to the revised manuscript.

We are thankful that the EIC and the reviewers have requested a number of additions and clarification, particularly regarding improvements to the presentation of the work and discussion of related literature. We have revised the manuscript carefully and addressed the comments raised by the reviewers. Meanwhile, we also provided a suitable rebuttal to each reviewer comment not addressed.

(3) Question: If you would like to make changes to your financial disclosure, please include your updated statement in your cover letter. Guidelines for resubmitting your figure files are available below the reviewer comments at the end of this letter.

Response: We are thankful to your comments and there are changes for financial disclosure. Funding projects has been listed in the revised manuscript and cover letter. We have carefully read the guidelines for resubmitting the figure files and believe that the figure file format should meet the requirements of the journal.

(4) Question: If applicable, we recommend that you deposit your laboratory protocols in protocols.io to enhance the reproducibility of your results. Protocols.io assigns your protocol its own identifier (DOI) so that it can be cited independently in the future. For instructions see: <http://journals.plos.org/plosone/s/submission-guidelines#loc-laboratory-protocols>. Additionally, PLOS ONE offers an option for publishing peer-reviewed Lab Protocol articles, which describe protocols hosted on protocols.io. Read more information on sharing protocols at <https://plos.org/protocols?utm_medium=editorial-email&utm_source=authorletters&utm_campaign=protocols>.
Response: We are thankful to the editor comments. The revised manuscript focuses the reclamation area of the 13 ports during 2002–2018, changes of coastline length, and reclamation stages. The findings have vital implications for scientifically regulating the spatial pattern and exploring the utility of port reclamation, and promoting regional sustainable development of coastal zones in dense port distribution. The revised manuscript has no laboratory analysis process and therefore, there is no laboratory protocols. Moreover, we have read the Submission Guidelines carefully in <https://journals.plos.org/plosone/s/submission-guidelines#loc-laboratory-protocols>. Thank you very much.

(5) Question: Journal Requirements:

When submitting your revision, we need you to address these additional requirements.

1. Please ensure that your manuscript meets PLOS ONE's style requirements, including those for file naming. The PLOS ONE style templates can be found at 
https://journals.plos.org/plosone/s/file?id=wjVg/PLOSOne_formatting_sample_main_body.pdf and 
<https://journals.plos.org/plosone/s/file?id=ba62/PLOSOne_formatting_sample_title_authors_affiliations.pdf>.

Response: We have read these additional requirements carefully and believe the revised manuscript should meet the additional requirements. Thank you very much.

(6) Question: We note that you have stated that you will provide repository information for your data at acceptance. Should your manuscript be accepted for publication, we will hold it until you provide the relevant accession numbers or DOIs necessary to access your data. If you wish to make changes to your Data Availability statement, please describe these changes in your cover letter and we will update your Data Availability statement to reflect the information you provide.

Response: We believe all the data demonstrated in the revised manuscript has been listed and the readers would access the data without any restriction.

(7) Question: We note that Figures 1, 3, 4 and 5 in your submission contain map/satellite images which may be copyrighted. All PLOS content is published under the Creative Commons Attribution License (CC BY 4.0), which means that the manuscript, images, and Supporting Information files will be freely available online, and any third party is permitted to access, download, copy, distribute, and use these materials in any way, even commercially, with proper attribution. For these reasons, we cannot publish previously copyrighted maps or satellite images created using proprietary data, such as Google software (Google Maps, Street View, and Earth). For more information, see our copyright guidelines: http://journals.plos.org/plosone/s/licenses-and-copyright.
 We require you to either (1) present written permission from the copyright holder to publish these figures specifically under the CC BY 4.0 license, or (2) remove the figures from your submission:
Response: We are thankful to the editor’s comments. The satellite images of Figure 1 were free to download from the website (<http://glovis.usgs.gov/>) and is freely available online. The Figure 1 was not produced from Google Maps and it was produced from the downloaded satellite. These satellite images are all open to the public on line and is allowed to access, download, copy, distribute in any way. Therefore, we believe Figures 1 has not copyrighted maps.

(8) Question: You may seek permission from the original copyright holder of Figures 1, 3, 4 and 5 to publish the content specifically under the CC BY 4.0 license.  
We recommend that you contact the original copyright holder with the Content Permission Form (http://journals.plos.org/plosone/s/file?id=7c09/content-permission-form.pdf) and the following text:
“I request permission for the open-access journal PLOS ONE to publish XXX under the Creative Commons Attribution License (CCAL) CC BY 4.0 (http://creativecommons.org/licenses/by/4.0/). Please be aware that this license allows unrestricted use and distribution, even commercially, by third parties. Please reply and provide explicit written permission to publish XXX under a CC BY license and complete the attached form.”
Please upload the completed Content Permission Form or other proof of granted permissions as an "Other" file with your submission.
In the figure caption of the copyrighted figure, please include the following text: “Reprinted from [ref] under a CC BY license, with permission from [name of publisher], original copyright [original copyright year].”
 Response: We are thankful to your constructive comments. We have contacted the original copyright holders to seek the allow to use the materials. These figures are freely distributed in website and we believe the Figures in the revised manuscript should meet the requirements of the journals.

(9) Question: If you are unable to obtain permission from the original copyright holder to publish these figures under the CC BY 4.0 license or if the copyright holder’s requirements are incompatible with the CC BY 4.0 license, please either i) remove the figure or ii) supply a replacement figure that complies with the CC BY 4.0 license. Please check copyright information on all replacement figures and update the figure caption with source information. If applicable, please specify in the figure caption text when a figure is similar but not identical to the original image and is therefore for illustrative purposes only. The following resources for replacing copyrighted map figures may be helpful: USGS National Map Viewer (public domain): <http://viewer.nationalmap.gov/viewer/> The Gateway to Astronaut Photography of Earth (public domain): <http://eol.jsc.nasa.gov/sseop/clickmap/> Maps at the CIA (public domain): https://www.cia.gov/library/publications/the-world-factbook/index.html and https://www.cia.gov/library/publications/cia-maps-publications/index.html
NASA Earth Observatory (public domain): http://earthobservatory.nasa.gov/
Landsat: <http://landsat.visibleearth.nasa.gov/> USGS EROS (Earth Resources Observatory and Science (EROS) Center) (public domain): http://eros.usgs.gov/#
Natural Earth (public domain): <http://www.naturalearthdata.com/>

Response: We are thankful to your constructive comments. We have known that you provided the following resources for replacing copyrighted map figures complies with the CC BY 4.0 license. Our figures are download from the website of USGS website and we believe it should meet your requirements.

(10) Question: We note that Figure 2 in your submission contain copyrighted images. All PLOS content is published under the Creative Commons Attribution License (CC BY 4.0), which means that the manuscript, images, and Supporting Information files will be freely available online, and any third party is permitted to access, download, copy, distribute, and use these materials in any way, even commercially, with proper attribution. For more information, see our copyright guidelines: http://journals.plos.org/plosone/s/licenses-and-copyright.
We require you to either (1) present written permission from the copyright holder to publish these figures specifically under the CC BY 4.0 license, or (2) remove the figures from your submission:
Response: The Figure 2 is taken from the corresponding author and we believe we have the copyright.

 (11) Question: You may seek permission from the original copyright holder of Figure 2 to publish the content specifically under the CC BY 4.0 license. 
We recommend that you contact the original copyright holder with the Content Permission Form (http://journals.plos.org/plosone/s/file?id=7c09/content-permission-form.pdf) and the following text:
“I request permission for the open-access journal PLOS ONE to publish XXX under the Creative Commons Attribution License (CCAL) CC BY 4.0 (http://creativecommons.org/licenses/by/4.0/). Please be aware that this license allows unrestricted use and distribution, even commercially, by third parties. Please reply and provide explicit written permission to publish XXX under a CC BY license and complete the attached form.”
Please upload the completed Content Permission Form or other proof of granted permissions as an "Other" file with your submission. 
In the figure caption of the copyrighted figure, please include the following text: “Reprinted from [ref] under a CC BY license, with permission from [name of publisher], original copyright [original copyright year].”
If you are unable to obtain permission from the original copyright holder to publish these figures under the CC BY 4.0 license or if the copyright holder’s requirements are incompatible with the CC BY 4.0 license, please either i) remove the figure or ii) supply a replacement figure that complies with the CC BY 4.0 license. Please check copyright information on all replacement figures and update the figure caption with source information. If applicable, please specify in the figure caption text when a figure is similar but not identical to the original image and is therefore for illustrative purposes only.

Response: The copyright of Figure 2 belongs to us and it was taken at July, 2019. At that time, the first author and the corresponding author conducted a field investigation of the landscape structure within the ports at the Bohai Rim

## Comments to the Author

(1) Question: Is the manuscript technically sound, and do the data support the conclusions? The manuscript must describe a technically sound piece of scientific research with data that supports the conclusions. Experiments must have been conducted rigorously, with appropriate controls, replication, and sample sizes. The conclusions must be drawn appropriately based on the data presented.

Reviewer #1: Yes

Reviewer #2: Yes

**Response:** Thank the reviewer’s **high evaluation** on the manuscript. We appreciate the reviewers' assessment to our manuscript. The reviewer thought that **the manuscript is technically sound and the data support the conclusion.**

Coastal land reclamation (CLR) has become a frequent approach to alleviate land shortages with the reclamation of port areas being the most prominent. The Bohai Sea is the most concentrated area of port reclamation worldwide. The manuscript applied the remote sensing images and field investigation to address the changes in different type coastlines and the CLR process of these ports in the reclaimed areas of the Bohai Sea. The port land reclamation experienced periods of acceleration, peak, deceleration, and stagnation since 2002, and the planned schedule of reclamation should be constrained. The findings have vital implications for scientifically regulating the spatial pattern and exploring the utility of port reclamation, and promoting regional sustainable development of coastal zones in dense port distribution. We believe that the revised manuscript has broad readers all over the world. The conclusion has been drawn appropriately based on the data presented.

(2) Question: Has the statistical analysis been performed appropriately and rigorously?

Reviewer #1: Yes

Reviewer #2: Yes

**Response:** Thank the reviewer’s **evaluation** on the manuscript. The manuscript has applied statistical analysis appropriately. We think that the current statements of the revised manuscript will meet the standard that the journal demands and the readers have no difficulties in understanding the content of the manuscript. We express our gratitude's to the reviewer for the helpful comments.

(3) Question: Have the authors made all data underlying the findings in their manuscript fully available?
The [PLOS Data policy](http://www.plosone.org/static/policies.action#sharing) requires authors to make all data underlying the findings described in their manuscript fully available without restriction, with rare exception (please refer to the Data Availability Statement in the manuscript PDF file). The data should be provided as part of the manuscript or its supporting information, or deposited to a public repository. For example, in addition to summary statistics, the data points behind means, medians and variance measures should be available. If there are restrictions on publicly sharing data—e.g. participant privacy or use of data from a third party—those must be specified.

Reviewer #1: Yes

Reviewer #2: Yes

**Response:** Thank the reviewer’s **evaluation** on the manuscript. We have known that the PLoS ONE Data policy requires the authors to make all data underlying the findings described in their manuscript fully available without restriction. The data presented in the manuscript has been provided as part of the manuscript. We express our gratitude's to the reviewer for the helpful comments.

(4) Question: Is the manuscript presented in an intelligible fashion and written in standard English?

PLOS ONE does not copyedit accepted manuscripts, so the language in submitted articles must be clear, correct, and unambiguous. Any typographical or grammatical errors should be corrected at revision, so please note any specific errors here.
Reviewer #1: Yes

Reviewer #2: Yes

**Response:** Thank the reviewer’s **evaluation** on the manuscript. The manuscript is presented in an intelligible fashion and written in standard English. More important, the writing expression has been revised by **a Native English Speaker** carefully from Elsevier. There is no any typographical or grammatically errors. Therefore, we believe the language in submitted article is clear, correct, and unambiguous. Please refer to the revised manuscript. Thank you.

(5) Question: Review Comments to the Author.

Please use the space provided to explain your answers to the questions above. You may also include additional comments for the author, including concerns about dual publication, research ethics, or publication ethics. (Please upload your review as an attachment if it exceeds 20,000 characters)

## Reviewer #1:

(1) Question: This paper tries to identify and solve in important question. The methodology is sound and the data seems valid. However, the paper provides too many pieces of information without effectively provide a reasonable link among them and explain how that information could be used in answering the research question and for better land reclamation and environmental regulation for the future of the study region. I have some major concerns listed below:
Information on funding and competing Interests should be placed at the end of the paper before reference list.
**Response:** Thank the reviewer’s **evaluation** on the manuscript. The reviewers think that the coastal port reclamation is an important question and the author tries to solve the crucial problem. Port reclamation is the main types of coastal reclamation. However, the spatial extent, percentages, and processes of these newly reclaimed ports are largely unknown. The reviewer think that the methodology is **sound and the data seems valid**. Thank the reviewer’s **evaluation** on the manuscript.

Moreover, we should not blame the port reclamation for several reasons. First, not all the reclaimed area is used as port construction and only 26.3% of reclaimed areas were applied for port construction in Tianjin and Tangshan ports.

Regarding with the link among the information, we have revised the manuscript and provided the research question for better land reclamation and environmental regulation for the future of the study region.

(2) Question: Introduction: very poor with limited literature about port cluster, coastal land reclamation, evolution stages, pattern optimization etc.
**Response:** Thank the reviewer’s **evaluation** on the manuscript. We have revised the manuscript in the Introduction and added some literature about port cluster, coastal land reclamation, evolution stages, pattern optimization. Please refer to the revised manuscript. Thank you again.

(3) Question: Study area and research method: very weak! How come we get to Table 1? And what is the justification of adopting equation 1-2? Source?

**Response:** We are thankful to the reviewer’s comments. The section study area and research method have been improved based on the reviewer’s comments. In the current version, some detailed information about study area have been added and the source of equation 1-2 have also been provided. Please refer to the revised manuscript.

(4) Question: Results: still simply copy paste from commissioned works.
**Response:** We are thankful to the reviewer’s comments. The Results section has been modified according to the reviewer’s comments.

(5) Question: Discussion: some interesting insights but still lack of coherency with the introduction/literature. The current section is not directly related to the analysis of this article.

**Response:** We are thankful to the reviewer’s comments. The reviewer thinks that the discussion has **some interesting insight**. As for the coherency with the introduction/literature, we have modified the discussion section and related information has been added in the revised manuscript.

(6) Question: Conclusion and policy implications: it mostly looks like a summary of the work done and the results obtained. Also, there should be some content in the conclusion regarding the limitations of the current research and future work possibilities.

**Response:** We are thankful to the reviewer’s comments. In the Conclusion and Discussion, we have added many sentences to illustrate the limitation of the current research and future work. We believe the revised conclusion should meet the requirements of the reviewers. Please refer to it.

(7) Question: There are more language and grammar issues than mentioned above. I highly recommend using a professional editor to correct them before re-submission.

**Response:** We are thankful to the reviewer’s comments. More important, the writing expression has been revised by **a Native English Speaker** carefully from Elsevier. There is no any typographical or grammatically errors. Therefore, we believe the language in submitted article is clear, correct, and unambiguous. Please refer to the revised manuscript.

(8) Question: Some literature to consult:
Demand prediction and regulation zoning of urban-industrial land: Evidence from Beijing-Tianjin-Hebei Urban Agglomeration, China. Environ Monit Assess 191, 412 (2019). https://doi.org/10.1007/s10661-019-7547-4
Understanding land surface temperature impact factors based on local climate zones, Sustainable Cities and Society (2021), doi: https://doi.org/10.1016/j.scs.2021.102818
Coupling Coordination Relationships between Urban-industrial Land Use Efficiency and Accessibility of Highway Networks: Evidence from Beijing-Tianjin-Hebei Urban Agglomeration, China. Sustainability. 2019; 11(5):1446.

**Response:** We are thankful to the reviewer’ comments. The three published articles have been added in the reference list. Please refer to it.

## Reviewer #2:

(1) Question: After a very careful reading of the work entitled "Land reclamation pattern and environmental regulation guidelines for port clusters in the Bohai Sea, China", I have found a very well-done work, well presented and organized, clear in concepts and methodology. The topic and context attract attention for many readers from various disciplines. The study is worth to be published in PLOS ONE after conducting the revisions.

**Response:** We are thankful to the reviewer’s **To Be Published** evaluation on the manuscript. The reviewer thinks that it is a **well-done work, well presented and organized, clear** in concepts and methodology. Meanwhile, the review believe the topic and context **attract attention** for many readers from various disciples. The manuscript takes the port reclamation in the Bohai Sea as an example to demonstrate the coastline change, percentage of port reclamation in all the reclaimed area. The manuscript is not to encourage the reclamation in coastal area, but to identify the truth of port reclamation. It has important implication for decision-makers to adopt reasonable reclamation policy in future. We express our gratitude to the kind reviewer for his constructive comments. Meanwhile, the reviewer has provided some comments to improve the quality of this manuscript.

(2) Question: Please further elaborate the innovation of the paper.

The language of this manuscript needs to be revised by native English speaking experts.

**Response:** We are thankful to the reviewer’s comments. The innovation of the paper has been proposed in the Conclusion and Abstract section. Moreover, the manuscript has been polished by a native English speaking expert. We believe the manuscript has been improved much in language.
