# Supplementary material for: Land reclamation pattern and environmental regulation guidelines for port clusters in the Bohai Sea, China
Source: PLoS One. 2021 Nov 3;16(11):e0259516. doi: 10.1371/journal.pone.0259516 (PMC8565771; doi:10.1371/journal.pone.0259516)
Supplement: Attachment — Submitted filename: Response to Reviewers20211008.docx [file pone.0259516.s002.docx]

## Responses to Editor-in Chief’s recommendation

(1) Question: Thank you for submitting your manuscript to PLOS ONE. After careful consideration, we feel that it has merit but does not fully meet PLOS ONE’s publication criteria as it currently stands. Therefore, we invite you to submit a revised version of the manuscript that addresses the points raised during the review process.

**Response:** We express our gratitude to you and the two anonymous reviewers for providing helpful suggestion in term of manuscript structure toward the revised manuscript. The Editor-in-Chief has made a decision of **merit evaluation and provided a revision opportunity for the manuscript**. The Editor-in-Chief maintain that the current status of the manuscript does not fully meet PLoS ONE's publication standard. These useful comments brought by the two reviewers are totally positive effective to the improvement of the revision version. We have addressed the reviewers' points one-by-one carefully based on the reviewer’s comments and we have submitted the second revised manuscript before the due date. We wish that the second revised manuscript would be accepted in the journal immediately.

(2) Question: Please submit your revised manuscript by Nov 04 2021 11:59PM. If you will need more time than this to complete your revisions, please reply to this message or contact the journal office at [plosone@plos.org](mailto:plosone@plos.org). When you're ready to submit your revision, log on to https://www.editorialmanager.com/pone/ and select the 'Submissions Needing Revision' folder to locate your manuscript file.
Please include the following items when submitting your revised manuscript:

- A rebuttal letter that responds to each point raised by the academic editor and reviewer(s). You should upload this letter as a separate file labeled 'Response to Reviewers'.
- A marked-up copy of your manuscript that highlights changes made to the original version. You should upload this as a separate file labeled 'Revised Manuscript with Track Changes'.
- An unmarked version of your revised paper without tracked changes. You should upload this as a separate file labeled 'Manuscript'.

**Response:** Very appreciated for your decision. The first author and the corresponding author will resubmit the second revised manuscript. The file that has been labeled as 'Response to Reviewers' has been prepared as a separate file and has been tackled to each inquiry proposed by the academic editor and the two anonymous reviewers. Additionally, we have prepared a separate file labeled "Revised Manuscript with Track Changes", which has identified the changes made to the original version. The unmarked changes have been also uploaded as a separated file. Please read the current revised manuscript.

We thank the EIC and the two reviewers for proposing a number of additions and clarification, particularly toward the improvements to the discussion of related literature. We have addressed the manuscript thoroughly and addressed the comments raised by the reviewers. At the same time, we also attached a rigorous rebuttal to each comment that has not been addressed by the two reviewers.

(3) Question: If you would like to make changes to your financial disclosure, please include your updated statement in your cover letter. Guidelines for resubmitting your figure files are available below the reviewer comments at the end of this letter.

**Response:** We are thankful to your constructive suggestion and there are no changes for financial disclosure. Because the funding information have been list in the previous revised manuscript and cover letter. We have carefully read the guidelines and we feel very strong that the format of the second revised manuscript should meet the criteria of the journal.

(4) Question: If applicable, we recommend that you deposit your laboratory protocols in protocols.io to enhance the reproducibility of your results. Protocols.io assigns your protocol its own identifier (DOI) so that it can be cited independently in the future. For instructions see: <http://journals.plos.org/plosone/s/submission-guidelines#loc-laboratory-protocols>. Additionally, PLOS ONE offers an option for publishing peer-reviewed Lab Protocol articles, which describe protocols hosted on protocols.io. Read more information on sharing protocols at <https://plos.org/protocols?utm_medium=editorial-email&utm_source=authorletters&utm_campaign=protocols>.
**Response:** We express our gratitude to the two anonymous reviewers for their constructive suggestions. The second version of revised manuscript highlights the reclamation status and reclamation stages, i.e., in area changes, especially of the coastline changes of the 13 ports during 2002–2018. The obtained results have important meanings for scientifically regulating the spatial pattern in coastal area, and proposing next reclamation policy in dense port distribution in the Bohai Rim area. The revised version has no laboratory analysis process and therefore, there is no laboratory protocols. Moreover, we have read the Submission Guidelines carefully in <https://journals.plos.org/plosone/s/submission-guidelines#loc-laboratory-protocols>. Thank you very much.

(5) Question: Please review your reference list to ensure that it is complete and correct. If you have cited papers that have been retracted, please include the rationale for doing so in the manuscript text, or remove these references and replace them with relevant current references. Any changes to the reference list should be mentioned in the rebuttal letter that accompanies your revised manuscript. If you need to cite a retracted article, indicate the article’s retracted status in the References list and also include a citation and full reference for the retraction notice.

**Response:** We are thankful to the EIC’s suggestion for the reference list. We have checked the reference list and believe the current reference list is complete and correct. If the reference has been changes, such as delete or replace, we will identify it in the rebuttal letter and the revised manuscript.

# References

1. Meng WQ, Hu BB, He MX, Liu BQ, Mo XQ, Li HY, et al. Temporal-spatial variations and driving factors analysis of coastal reclamation in China. Estuar Coast Shelf Sci. 2017; 191: 39–49.

2. Yang J, Ren JY, Sun DQ, Xiao XM, Xia JH, Jin C, et al. Understanding land surface temperature impact factors based on local climate zones. Sustain Cities Soc. 2021; 69:102818. https://doi.org/10.1016/j.scs.2021.102818.

3. Zuo TL, Zha YP, Nei XJ, Li R, Qi Y, Dong M. Research on planning concept of ecological port. Port Waterw. Eng. 2017; 5: 56–61 (in Chinese).

4. Izaguirre C, Losada IJ, Camus P, Vigh JL, Stenek V. Climate change risk to global port operations. Nat Clim Change. 2021; 11: 14–20. https://doi.org/10.1038/s41558-020-00937-z.

5. Martínantón M, Negro V, Campo JMD, Lópezgutiérrez JS, Esteban MD. Review of coastal land reclamation situation in the world. J. Coast Res SI. 2016; (75): 667–671.

6. Hanson SE, Nicholls RJ. Demand for ports to 2050: climate policy, growing trade and the impacts of sea-level rise. Earth’s Future. 2020; http://doi.org/10.1029/2020EF001543.

7. Chen YY. Survey on influence of maritime port cluster effect on offshore regional economy based on grey correlation model. J. Coast Res 2019; 94: 707-711. Doi:10.2112/SI94–140.1.

8. Chen YP, Wei YQ, Peng LH. Ecological technology model and path of seaport reclamation construction. Ocean. Coastal. Manage. 2018; 165: 244–257. https://doi.org/10.1016/j.ocecoaman.2018.08.031.

9. Pang H, Dong SH. Inventory collaboration in coastal cluster supply chain. J of Coastal Res. 2019; 94: 617–620.

10. Wang W, Liu H, Li YQ, Su JL. Development and management of land reclamation in China. Ocean Coastal Manage. 2014; 102: 415–425.

11. Xu JY, Zhang ZX, Zhao XL, Wen QK, Zuo LJ, Wang X, et al. Spatial-temporal analysis of coastline changes in northern China from 2000 to 2012. Acta Geograph. Sin. 2013; 68(5): 651–660.

12. Sengupta D, Chen RS, Meadows ME. Building beyond land: an overview of coastal land reclamation in 16 global megacities. Appl Goegr. 2018; 90: 229–238.

13. Chen WG, Wang DC, Huang Y, Chen LD, Zhang LH, Wei XW, et al. Monitoring and analysis of coastal reclamation from 1995–2015 in Tianjin Binhai New Area, China. Sci Rep. 2017; 7: 3850.

14. Tian B, Wu WT, Yang ZQ, Zhou YX. Drivers, trends, and potential impacts of long-term coastal reclamation in China from 1985 to 2010. Estuar Coast Shelf Sci. 2016; 170: 83–90.

15. Wahl T, Haigh ID, Nicholls RJ, Arns A, Dangendorf S, Hinkel J, et al. Understanding extreme sea levels for broad-scale coastal impact and adaptation analysis. Nature Communication. 2017; 8:16075.

16. Li CX, Gao X, He BJ, Wu JY, Wu KN. 2019. Coupling coordination relationships between urban-industrial land use efficiency and accessibility of highway networks: evidence from Beijing-Tianjin-Hebei Urban agglomeration, China. Sustainability. 11: 1446, doi:10.3390/su11051446.

17. Li JG, Pu LJ, Zhu M, Zhang J, Li P, Dai XQ, et al. Evolution of soil properties following reclamation in coastal areas: a review. Geoderma. 2014; 226-227(1): 130–139.

18. Piersma T. Threats to intertidal soft-sediment ecosystems. University of Groningen. 2009

19. Jaramillo E, Lagos NA, Labra FA, Paredes E, Acuna E, Melnic D, et al. Recovery of black-necked swans, macrophytes and water quality in a Ramsar wetland of southern Chile: assessing resilience following sudden anthropogenic disturbances. Sci. Total Environ. 2018; 628–629, 291–301.

20. UNEP, 2016. Population within 100 Kilometers of Coast. Available at <http://geodata.grid.unep.ch/options.phs?selectedID=280&selectedDatasettype=1>. (Accessed 16 May 2016)

21. Zhu GR, Xie ZL, Xu XG. The landscape change and theory of orderly reclamation sea based on coastal management in rapid industrialization area in Bohai Bay, China. Ocean Coastal Manage. 2016; 133, 128–137.

22. Chee SY, Othman AG, Sim YK, Adam ANM, Firth LB. Land reclamation and artificial islands: walking the tightrope between development and conservation. Glob. Ecol Conserv. 2017; 12: 80–95.

23. Kim RH, Kim JH, Ryu JS, Koh DC. Hydrogeochemical characteristics of groundwater influenced by reclamation, seawater intrusion, and land use in the coastal area of Yeonggwang, Korea. Geosci J. 2019; 23: 603–619.

24. Lu QQ, Bai JH, Zhang GL, Wu JJ. Effects of coastal reclamation history on heavy metals in different wetland type soils in the Pearl River Delta: levels, sources and ecological risks. J of Clean Prod. 2020; 272: 122668.

26. Bulleri F, Chapman MG. The introduction of coastal infrastructure as a driver of change in marine environments. J Appl Ecol. 2010; 47(1): 26–35.

27. Guneroglu N, Acar C, Guneroglu A, Dihkan M. Coastal land degradation and character assessment of Southern Black Sea landscape. Ocean. Coastal Manag. 2015; 118: 282–289.

28. Jongman B, Ward PJ, Aerts JC. Global exposure to river and coastal flooding: long term trends and changes. Global Environment Change. 2012; 22(4): 823–835.

29. Cheng ZX, [Jalon-Rójas](https://www.sciencedirect.com/science/article/abs/pii/S0272771419309874#!) I, [Wang](https://www.sciencedirect.com/science/article/abs/pii/S0272771419309874#!) XH, [Liu](https://www.sciencedirect.com/science/article/abs/pii/S0272771419309874#!) Y. Impacts of land reclamation on sediment transport and sedimentary environment in a macro-tidal estuary. Estuar Coast Shelf Sci. 2020; 242: 106861.

30. Wu WT, Yang ZQ, Tian B, Huang Y, Zhou YX, Zhang T. Impacts of coastal reclamation on wetlands: loss, resilience, and sustainable management. Estuar. Coast Shelf Sci. 2018; 210:153–161.

31. Xu Y, Cai YP, Sun T, Tan Q. A multi-scale integrated modeling framework to measure comprehensive impact of coastal reclamation activities in Yellow River estuary, China. Mar Pollut Bull. 2017; 122(1-2): 27–37.

32. Suo AN, Wang P, Yuan DW, Yu YH, Zhang MH. Study on monitoring and analysis of existing sea reclamation resource based on high resolution satellite remote sensing imagery- a case in south coast of Yingkou. Acta Oceanol Sin. 2016; 38(9): 54-63 (in Chinese).

33. Peng B, Xie H, Xie Z, Li XL, Yang RG. Sea reclamation dynamic change and driving factors analysis within a port. Geospatial Infor. 2019; 3: 109–112.

34. Wei F, Han GX, Han M, Zhang JP, Li YZ, Zhao J. Temporal-spatial dynamic evolution and mechanism of shoreline and the sea reclamation in the Bohai Rim during 1980-2017. Scien Geogr Sin. 2019; 39(6), 997–1007.

35. Yin YH. Thinking on large area reclamation in Caofeidian, Tangshan city, Hebei province. Mar Geol Lett. 2007; 3: 1–10 (in Chinese).

36. Duan HB, Zhang H, Huang QF, Zhang YK, Hu MW, Niu YN, et al. Characterization and environmental impact analysis of sea land reclamation activities in China. Ocean Coast Manage. 2016; 130: 128–137.

37. Pelling HE, Uehara K, Green JAM. The impact of rapid coastline changes and sea level rise on the tides in the Bohai Sea, China. J. Geophys. Res: Oceans. 2013; 118(7): 3462–3472.

38. Shen CC, Shi HH, Zheng W, Li F, Peng ST, Ding DW. Study on the cumulative impact of reclamation activities on ecosystem health in coastal waters. Mar Pollut Bull. 2016; 103: 144–150.

39. Fu YB, Cao K, Wang F, Zhang FS. Research of quantitative assessment method of reclamation intensity and potentiality. Ocean Development and Management. 2010. 27(1): 27–30.

40. Pang RZ. Dynamic evaluation of main sea ports in mainland China based on DEA model. Economic Research Journal. 2006.(6): 92–100.

41. Xu N. Research on spatial and temporal variation of China mainland coastline and coastal engineering. University of Chinese Academy of Sciences. 2016.

42. Li S, Zhao S, Zhou X, Li H. Analysis on environment of Changlu salt pan area in North China. Tianjin Sci. Technol. 2011, 5: 114–116 (in Chinese).

43. Jin YD, Zhang QF, Li XB, Wang LN, Ye FJ. Effects of Tianjin reclamation projects on the Bohai Bay water exchange. Mar. Sci. Bull. 2017, 36(5): 578–584.

44. Xiao QC, Wei YS, Wang YW. Driving factors of coastal wetland degradation in Binhai New Area of Tianjin. Acta Sci. Circumst. 2012; 2: 480–488 (in Chinese).

45. Yue Q, Zhao M, Yu HM, Xu W, Qu L. Total quantity control and intensive management system for reclamation in China. Ocean Coast Manage. 2016, 120, 64–69.

46. Yue Q, Zhao M, Yu HM, Xu W, Ou L. Total quantity control and intensive management system for reclamation in China. Ocean Coast Manag. 2016; 120: 64–69. http://dx.doi.org/10.1016/j.ocecoaman.2015.11.026.

47. Hearly MG, Hickey KR. Historic land reclamation in the intertidal wetlands of the Shannon estuary, western Ireland. J Coast. Res. 2002, S36: 365–373.

48. UNEP. Marine and Coastal Ecosystems and Human Well-being: a Synthesis Report Based on the Findings of the Millennium Ecosystem Assessment. UNEP. 2006.

49. GOSC. State Council executive meeting decides to further implement the key industry adjustment and revitalization plan (in Chinese). 2010. Available in the website. http://www.gov.cn/ldhd/2010-02/24/content_1540570.html.

50. Suo AN, Zhang MH. Sea areas reclamation and coastline change monitoring by remote sensing in coastal zone of Liaoning in China. J Coastal Res. 2015; 73:725-729.

51. Li CX, Gao X, Wu JY, Wu KN. Demand prediction and regulation zoning of urban-industrial land: evidence from Beijing-Tianjin-Hebei urban agglomeration, China. Environ Monit Assess. 2019; 191: 412, <https://doi.org/>10.1007/ s10661-019-7547-4.

52. Cui BS, He Q, Gu BH, Bai JH, Liu XH. China’s coastal wetlands: understanding environmental human impacts for management and conservation. Wetlands. 2016; 36: S1–S9.

53. Sousa CAM, Cunha ME, Ribeiro L. Tracking 130 years of coastal wetland reclamation in Ria Formosa, Portugal: opportunities for conservation and aquaculture. Land Use Policy. 2020; 94: 104544. <https://doi.org/10.1016/j.landusepol.2020.104544>.

54. Arkema KK, Guannel G, Verutes G, Wood SA, Guerry A, Ruckelshaus M, et al. Coastal habitats shield people and property from sea-level rise and storms. Nat. Clim. Change. 2013; 3: 913–918.

55. Currin C, Davis J, Baron LC, Malhotra A, Fonseca M. Shoreline Change in the New River Estuary, North Carolina: Rates and Consequences. J Coastal Res. 2015; 31(5): 1069–1077.

56. Dafforn KA, Mayer-Pinto M, Morris RL, Waltham. Application of management tools to integrate ecological principles with the design of marine infrastructure. J Environ. Manage. 2015; 158: 61–73.

57. Zhu GR, Xu XG, Wang H, Li TY, Feng Z. The ecological cost of land reclamation and its enlightenment to coast sustainable development in the northwestern Bohai Bay, China. Acta Oceanol. Sin. 2017; 36(4):97–104.

58. Zhang YZ, Chen RS, Wang Y. Tendency of land reclamation in coastal areas of Shanghai from 1998 to 2015. Land Use Policy. 2020; 91: 104370.

59. Zhang BaL. Environmental impacts of sea reclamation in Jiaozhou Gulf, Shandong province of China. Natural Hazards. 2012; 63: 1269-1272. Doi: 10.1007/s11069-012-0157-x.

60. Nicholls RJ, Wong PP, Burkett VR, Codignotto JO, Hay JE, McLean RF, et al. Coastal systems and low-lying areas. Climate change 2007: impacts, adaptation and vulnerability. In: Parry ML, Canziani OF, Palutikof JP, van der Linden PJ, Hanson CE (Eds.), Contribution of Working Group II to the Fourth Assessment Report of the Intergovernmental Panel on Climate Change. Cambridge University Press, Cambridge, UK, 2007; 315–356.

61. Ramesh R, Chen Z, Cummins V, Day J, D’Elia C, Dennison B, et al. Land-ocean interactions in the coastal zone: past, present & future. Anthropocene. 2015; 12: 85–98.

62. Han X. 2011 National Economic and Social Development Bulletin of China’s 11 Coastal Provinces and Metropolises (in Chinese). 2012. Available in the website: <http://district.ce.cn/zg/201202/27t20120227_23109765.shtml>.

Additional Editor Comments (if provided):

(1) Question: The layout of the paper is very bad, especially the tables and headings.

**Response:** We are thankful to the Editors comments. The editor thinks the layout of the manuscript is bad and we have made improvements in tables and headings. Please refer to the revised manuscript.

(2) Question: I suggest the results should be better discussed and justified, such as whether they are consistent with previous studies or analyzing the reasons for the empirical results. At present, there are many contents in the discussion section that are not closely related to this paper.

**Response:** We are thankful to the editor’s comment. The editor thinks the discussion section is not consistent with the results and we have modified the discussion based on the editor’s comments. Please refer to the revised manuscript.

## Comments to the Author

(1) Question: If the authors have adequately addressed your comments raised in a previous round of review and you feel that this manuscript is now acceptable for publication, you may indicate that here to bypass the “Comments to the Author” section, enter your conflict of interest statement in the “Confidential to Editor” section, and submit your "Accept" recommendation.

Reviewer #1: (No Response)

Reviewer #2: All comments have been addressed

**Response:** Thank the reviewer’s **high evaluation** on the manuscript. The second reviewer believe that **‘All comments in the manuscript have been addressed**’. We totally agree with the reviewer’s comments. During previous revision process, we took plenty of time revise the manuscript. Each comments proposed by the two reviewers has been tackled correctly. Coastal land reclamation has become a common method to mitigate land shortages in coastal area. The Bohai Sea is the most concentrated area of port reclamation worldwide. We believe that the revised manuscript has broad readers worldwide.

(2) Question: Is the manuscript technically sound, and do the data support the conclusions? The manuscript must describe a technically sound piece of scientific research with data that supports the conclusions. Experiments must have been conducted rigorously, with appropriate controls, replication, and sample sizes. The conclusions must be drawn appropriately based on the data presented

Reviewer #1: (No Response)

Reviewer #2: Yes

**Response:** We are thankful to the two reviewers for the helpful comment. The Reviewer #2 think that **the manuscript technically sound and the data support the conclusion**. The revised manuscript used the Landsat remote sensing images and field investigation to address the changes in different type coastlines and the CLR process of these ports in the reclaimed areas of the Bohai Sea. The port land reclamation experienced periods of acceleration, peak, deceleration, and stagnation since 2002, and the planned schedule of reclamation should be constrained. The results obtained in the manuscript have important implications for further scientifically reclamation, and promoting regional sustainable development of coastal zones in dense port distribution.

(3) Question: Has the statistical analysis been performed appropriately and rigorously?

Reviewer #1: (No Response)

Reviewer #2: Yes

**Response:** We are thankful to the reviewer’s evaluation on the manuscript. The revised manuscript has used the statistical method to identify the reclamation status appropriately. We believe the latest revised manuscript should meet the requirements of the journal. The readers will have no difficulties in understanding the ideas of the manuscript. We express our gratitude's to the two reviewer for their helpful comments.

(4) Question: Have the authors made all data underlying the findings in their manuscript fully available?
The [PLOS Data policy](http://www.plosone.org/static/policies.action#sharing) requires authors to make all data underlying the findings described in their manuscript fully available without restriction, with rare exception (please refer to the Data Availability Statement in the manuscript PDF file). The data should be provided as part of the manuscript or its supporting information, or deposited to a public repository. For example, in addition to summary statistics, the data points behind means, medians and variance measures should be available. If there are restrictions on publicly sharing data—e.g. participant privacy or use of data from a third party—those must be specified.

Reviewer #1: (No Response)

Reviewer #2: Yes

**Response:** We are thankful the reviewer’s assessment toward the manuscript. We have known that the PLoS ONE Data policy requires the authors to make all data underlying the findings described in their manuscript fully available without restriction. All the images data are free download in the website. Therefore, the data presented in the manuscript has been provided as part of the manuscript. We express our gratitude's to the reviewer for the helpful comments.

(5) Question: Is the manuscript presented in an intelligible fashion and written in standard English? PLOS ONE does not copyedit accepted manuscripts, so the language in submitted articles must be clear, correct, and unambiguous. Any typographical or grammatical errors should be corrected at revision, so please note any specific errors here.
Reviewer #1: (No Response)

Reviewer #2: Yes

**Response:** We are thankful to the reviewer’s **assessment** toward the manuscript. The manuscript is presented in an intelligible fashion and written in a standard expression. The writing expression has been revised by **a Native English Speaker** carefully. There is no any typographical or grammatically errors. Therefore, we believe the language in current version is clear, correct, and unambiguous. Please refer to the revised manuscript. Thank you again.

## Review Comments to the Author.

Please use the space provided to explain your answers to the questions above. You may also include additional comments for the author, including concerns about dual publication, research ethics, or publication ethics. (Please upload your review as an attachment if it exceeds 20,000 characters)

### Reviewer #1:

(1) Question: Although the author has done a lot of revision work, there are still many problems and I cannot recommend it for publication at the present version.
1. The layout of the paper is very bad, especially the tables and headings.

**Response:** We are thanking the reviewer’s **evaluation** on the manuscript. The reviewers said that **the author has done a lot of revision work**. However, there are still many problems that need further revision. Port reclamation is the main types of coastal reclamation. However, we should not blame the port reclamation for several reasons. First, not all the reclaimed area is used as port construction and only 26.3% of reclaimed areas were used as port construction in the Bohai Sea.

The reviewer think that the layout of the paper is bad, especially the tables and headings. We have modified the Tables and Headings based on the reviewer’s comments. Please refer to the revised manuscript.

(2) Question: I suggest the results should be better discussed and justified, such as whether they are consistent with previous studies or analyzing the reasons for the empirical results. At present, there are many contents in the discussion section that are not closely related to this paper.

**Response:** We are thankful to the reviewer’s comments. The reviewer thinks that the results should be better discussed and justified. The discussion section in the revised manuscript is consistent with previous studies. As for the coherency with the discussion, we have modified the discussion section and related information has been added in the revised manuscript. Some information that is not closely related to the paper has been deleted in the revised manuscript.

### Reviewer #2:

(1) Question: This manuscript has been revised in detail according to the comments of the reviewers. The current version is worth publishing.

**Response:** We are thankful to the reviewer and the reviewer think the current version is **worth publishing**. The reviewer thinks that **the manuscript has been revised in detail according to the comments of the reviewers**. The revised manuscript takes the Bohai Sea as an example to demonstrate the coastline length change, changes of area percentage of port reclamation within the port boundary of different ports. The purpose of the revised manuscript is not to agree with the reclamation in China, but to identify the area percentage of port reclamation, i.e., used for port purpose, in the reclaimed area. The manuscript has vital meanings for decision-makers to revise the reclamation policy in future. The ports will play an increasingly important role in the world due to the frequent exchanges of materials worldwide. We express our gratitude to the warm-hearted reviewer for the constructive comments.
